# A comparison of patient self-referrals and general practitioner referrals to the emergency department in Ireland during the COVID-19 pandemic: secondary data analysis of the “Better Data, Better Planning” study

**DOI:** 10.1007/s11845-026-04320-3

**Published:** 2026-05-07

**Authors:** Muhammad Shan, Rose Galvin, Louise A. Barry, Collette Devlin, Gillian Corey, Fergal Cummins, Damien Ryan, Niamh M. Cummins

**Affiliations:** 1 School of Medicine, Faculty of Education and Health Sciences, SLÁINTE Research and Education Alliance in General Practice, Primary Healthcare and Public Health, University of Limerick, Limerick, Ireland; 2 General Internal Medicine, Tipperary University Hospital, Tipperary, Ireland; 3 School of Allied Health, Faculty of Education and Health Sciences, Ageing Research Centre, Health Research Institute, University of Limerick, Limerick, Ireland; 4 Ageing Research Centre, Health Research Institute, University of Limerick, Limerick, Ireland; 5 Department of Nursing and Midwifery, Faculty of Education and Health Sciences, University of Limerick, Limerick, Ireland; 6 ALERT Limerick EM Education Research Training, Emergency Department, University Hospital Limerick, Limerick, Ireland; 7 Department of Paramedicine, Faculty of Medicine, Nursing and Health Sciences, Monash University, Melbourne, VIC Australia

**Keywords:** General practitioner, Emergency department, Crowding, Alternative care pathways, Health seeking behaviour

## Abstract

**Background:**

Internationally crowding in Emergency Departments (ED) has become a public health issue. In Ireland, primary care is centred around General Practitioners who can refer patients with acute illnesses or injuries to the ED. Patients can also choose to go directly to the ED if they require emergency care and self-referrals to the ED have been rising in recent years.

**Aim:**

The study aims to describe the demographic and clinical characteristics of patients who self-referred compared to patients referred by GPs in Irish EDs during the COVID-19 pandemic.

**Methods:**

This was a secondary data analysis of a cross-sectional, multi-centre study describing Irish ED utilisation; the Better Data, Better Planning (BDBP) Study. Descriptive comparisons were made between GP referrals to the ED and self-referrals in relation to; demographics, clinical data, healthcare utilisation, service awareness and other factors, with data being analysed in SPSS.

**Results:**

Demographic differences were minimal however significant differences were observed between GP referrals and self-referrals for; types of clinical presentations (*p* < 0.05), duration of complaint (*p* < 0.001), awareness of other services (*p* < 0.05), need for reassurance (*p* < 0.001), acquiring a second opinion (*p* < 0.05) and utilisation of GPs over the year (*p* < 0.05).

**Conclusion:**

Demographics were similar between GP referrals and self-referrals to the ED however significant behavioural and clinical differences were observed across groups. Awareness of these factors could be useful for policymakers in service planning for ED crowding generally and also more specifically in the event of a future pandemic.

## Background

Acute healthcare systems depend heavily on Emergency Departments (ED) [1]. An ED is intended to provide “rapid, high-quality, continuously accessible, unscheduled care” to patients suffering from a wide range of acute injuries and illnesses [2]. Crowding in ED has emerged as a public health issue with periods of mismatch between the demand for services in ED and the capacity to meet that demand in an appropriate time-frame. This leads to long wait times and delays in the provision of emergency care. Crowding in the ED is associated with negative clinical outcomes, including a higher death and complication rate [3]. Patient flow metrics (such as wait times, visit lengths, and the proportion of patients seen within their triage target time are frequently used to assess ED system performance. This is a very complex issue which involves balancing production and demand [4]. Internationally, hospital bed capacity is a significant factor that contributes to crowding in the ED creating an “access block” for admitted patients. This is particularly the case in Ireland which has one of the lowest inpatient beds per capita rate and the highest occupancy rates in the OECD region and across Europe [5].

Crowding in the ED has been characterized as a fear both for patient safety and public health around the world [6]. Many countries, such as Ireland, Canada and Australia report significant and unsustainable increases in ED presentations. However, evidence suggests that these increases cannot be attributed solely to population growth [7, 8]. Avoidable or non-urgent presentations to the ED can contribute to crowding [9]. Non-urgent presentations account for a significant portion of ED demand in several countries; for example, in the United States and Canada, these patients comprise 9% to 60% of the demand, while in Europe, they account for 19.6% to 40.9% [3]. Non-urgent ED visits are difficult to address because hospitals cannot prevent patients from presenting to the ED directly, and non-urgent patients frequently prefer ED to general practitioners (GPs) due to perceptions of severity of illness or injury, and the belief that the ED is better equipped to handle their care [10].

Primary care is centred around GPs in many healthcare systems [11] with onward referrals to secondary care, including the ED [12]. In Ireland GP referrals comprise 37% of ED attendances [13]. In Sweden it has been reported that 13% of ED attendances result from GP referrals [14], compared to 8% in Germany [15]. While it must be acknowledged that these international examples differ from the Irish system, it would be of interest to further examine the characteristics of GP referrals to EDs nationally. This work is topical due to the GP shortage in Ireland [16] meaning that that self-referrals to the ED will rise in the future.

Health seeking behaviour for urgent and emergency care in Ireland during the context of the COVID-19 pandemic also warrants further exploration. International evidence suggests that patients did forgo care in the early months of the pandemic, which in the United States included cancellation of general medical appointments and preventative care visits [17]. Also, while an initial fall in ED attendances was observed during lockdown periods in Scotland, these services did return to historical levels within a period of months [18]. In Ireland, within the Better Data, Better Planning (BDBP) study, an analysis of presentations during the pandemic at University Hospital Limerick found that 10% of patients presented to the ED because their GP was unable to provide care due to COVID-19 restrictions [19].

## Aim

The primary aim of this study is to describe the demographic and clinical characteristics of patients who self-refer to EDs in comparison to patients referred by GPs in the Irish setting during the COVID-19 pandemic. A secondary aim is to describe the factors influencing attendance to the ED from the patient perspective.

## Methods

### Setting

In Ireland, GPs are typically the first point of contact for patients with a health issue or illness that does not require emergency treatment and are responsible for referrals to emergency or specialist treatment when required. However, the Irish structure of funding of primary care does mean that there can be issues gaining access to GP services. Almost half of the population (43%) have free access to GP services because they hold a full medical card with a further 11% holding a GP visit card [20] but the remainder fall under the category of private patients and pay full fees for GP services [21].

Ireland’s public hospital structure is categorised into seven hospital groups: Royal College of Surgeons in Ireland (RCSI) Hospital Group, Ireland East Hospital Group, Dublin Midland Hospital Group, South/Southwest Hospital Group, University of Limerick Hospital Group, Children’s Health Ireland, and Saolta Hospital Group. These hospitals are working to provide unscheduled/emergency care, Maternity services, Diagnostics, Inpatient and Outpatient care services [22].

The BDBP study was a census study conducted in regional EDs at academic teaching hospitals in urban and rural areas across Ireland in 2020. Several factors were considered in selecting hospital sites, including geographic location, urban/rural mix, and socioeconomic status. Among the seven hospital groups, representatives of five hospitals were engaged in the study, University Hospital Limerick (UHL); University of Limerick Hospitals, St. Vincents University Hospital (SVUH); Ireland East Hospital Group, University Hospital Kerry (UHK); Southwest Hospital Group, Midlands Regional Hospital Tullamore (MRHT) and St. James University Hospital (SJUH); Dublin Midlands Hospital Group. According to the inclusion criteria, only adult participants were selected consequently CHI Hospital Group did not qualify for inclusion in this study.

### Design

This was a secondary data analysis of a cross-sectional, multi-centre study describing the demography and clinical characteristics of Irish ED patients, the BDBP Study [23].

### Participants and procedure

A potential recruitment pool consisted of all adult patients attending each ED during the sampling period and to account for diurnal and seasonal variations in attendance patterns, data were collected over separate 24-hour periods at each site over a one year period; January (MRHT), February (UHL) September (SVUH), November (SJUH) and December (UHK). Each census took place from Thursday at 12 pm to Friday at 12 pm. In most Irish and UK EDs, the Manchester Triage System (MTS) is used to determine the severity of a case, based on presenting symptoms and signs. Patients are categorised on the basis of urgency to required treatment i.e., Immediate, Very Urgent, Urgent, Standard, and non-urgent [24]. This study was conducted in accordance with the following inclusion criteria; (A) Adults ≥ 18 (B) Medically stable MTS (C) Patients who were capable of giving informed consent. The exclusion criteria applied were as follows; (A) Patients presenting with mental health disorders (B) Scheduled admissions to the ED (C) Patients who were unable to communicate due to English language barrier (D) Patients with altered capacity due to drugs or alcohol inebriation deemed by the triage nurse to be unfit for consent and interview. Suspected and confirmed patients with COVID-19 were also engaged in this study and Infection Prevention and Control protocols were strictly followed by the research team. Patients who were COVID-19 coronavirus positive or suspected of having the viral infection were recruited in the study However, confirmation of COVID-19 diagnosis was not included in BDBP due to differences in local testing protocols and reporting across sites. Specific exclusion criteria for this secondary data analysis pertains to patients who were transported to the hospital by emergency ambulance, regardless of method of referral.

In addition to completing a self-report questionnaire, participants consented to the sharing of their medical records including clinical presentation, triage classification, demographics, Health insurance, duration of stay and disposition. Collaboration between emergency medicine clinicians and researchers led to the development of the questionnaire, the recruitment rate differed site to site and ranged from 75 to 100% of eligible presentations. The questionnaire was designed to include open-ended answers, multiple choice questions (MCQs) and rating scales. The following categories were analysed in the questionnaire; Demography, health care utilisation in last 12 months, GP consultation prior to attending ED and factors leading to the ED visit. In this study, socioeconomic status was based on electoral divisions, which are the smallest geographically defined regions in the State for which Small Area Population Statistics (SAPS) are released each year. Adjacency to the health services i.e., Distance from ED and GP was self-described by the participants in kilometres but not validated by address which is a limitation of this data. The full protocol for the BDBP study has previously been described [23].

### Data analysis

SPSS (IBM SPSS Statistics Version 28.0.1 0.1) was used for all data analysis. Descriptive statistics were used for calculation of demographics and clinical characteristics. The Kolmogorov- Smirnov test was used for normality testing. Categorical variables were described as frequencies and percentages and Pearson chi-square test was used to explore the relationship between variables. Furthermore, to determine where the difference lies between different categories post hoc analysis was performed using the Bonferroni method. As data from the continuous variables was non-parametric it is listed as Median (Interquartile Range; IQR) according to the distribution. The Mann-Whitney test was used to compare the two groups. *P* < 0.05 is considered statistically significant.

## Results

### Study population and demographics

A total of 249 patients met the inclusion criteria for the study. The median age for the cohort was 50y (IQR 34–65) and the range was 18-100y (Table 1). Regarding gender, females comprised 52% of the cohort while males comprised 48%. In relation to civil status, the majority of the participants reported being in relationship (*n* = 155, 64%) and residential status indicated that over half of the participants shared a residence with a partner (*n* = 130, 53%). Secondary education was the highest education level obtained (*n* = 103, 42%) and the most frequently reported occupational status was the employee category (*n* = 107, 43%). With regard to socioeconomic status 40% of participants were living below average means (*n* = 92). The majority of patients attended ED as public patients holding a medical card (*n* = 105, 53%). Participants living within 5 km of their General Practitioner (GP) constituted the majority of patients (*n* = 138, 59%) and 42% lived within 10 km from ED (*n* = 102). The mode of transport category showed that 82% of participants travelled to the ED by private car.

When comparisons were made between patients who were referred to the ED by a GP (*n* = 147) and patients who self-referred (*n* = 102), there was no statistical difference recorded across the groups regarding gender or age. The median patient age was 50y (IQR = 35–65) for those who were referred by GP and 47y (IQR = 32–65) for self-referred participants. There were also no differences between GP referrals and self-referrals with regard to civil status, residential status, principal status, socioeconomic status, health care coverage and distance to a GP surgery (Table 1).

The differences between groups did approach significance in relation to both educational status (*p* = 0.085) and distance to the ED (*p* = 0.085), with GP referrals living farther away from the ED than self-referrals. A significant difference was recorded between groups regarding mode of transport with patients referred by a GP more likely to travel using private transport (GP referral 88% vs. self-referral 73%; *p* < 0.01).

**Table 1:** Sociodemographic characteristics for GP referrals and self-referrals to the ED (*n* = 249)

Category	Variable	TOTAL *n* = 249 %	GP referral *n* = 147 %	Self-referral *n* = 102 %	*P* value
Gender	Female Male	129 (52%) 120 (48%)	82 (56%) 65 (44%)	47 (46%) 55 (54%)	0.132
Age Age Category	Median, IQR Range 18-39y 40-64y 65y+	50, 34–65 18–100 90 96 63	50, 35–65 18–92 51 59 37	47, 32–65 19–100 39 37 26	0.968 0.801
Civil Status	Partner/Married Separated/Divorced Widowed Single	155 (63%) 16 (7%) 19 (8%) 55 (22%)	99 (68%) 10 (7%) 9 (6%) 27 (19%)	56 (56%) 6 (6%) 10 (10%) 28 (28%)	0.179
Residential Status	Partner Family Lives Alone Other e.g. co-share	130 (53%) 65 (26%) 38 (15%) 13 (5%)	81 (56%) 40 (27.6%) 18 (12.4%) 6(4.1%)	49 (48%) 25 (25%) 20 (20%) 7 (6%)	0.287
Principal Status	Employee Self-employed Family Carer Retired Unemployed Student	107 (43%) 23 (9%) 22 (9%) 61 (25%) 22 (9%) 12 (9%)	62 (43%) 12 (8%) 16 (11%) 37 (25%) 10 (7%) 8 (6%)	45 (44%) 11 (11%) 6 (6%) 24 (23%) 12 (12%) 4 (4%)	0.519
Education	No formal education Primary Secondary Technical/Vocational Third Level	3 (1%) 21 (9%) 103 (42%) 45 (18%) 73 (30%)	1 (1%) 18 (13%) 58 (41%) 27 (19%) 39 (27%)	2 (2%) 13 (3%) 45 (44%) 18 (18%) 34 (33%)	0.085
Socioeconomic Status	Affluent Above Average Below Average Disadvantaged Very Disadvantaged	41 (18%) 73 (32%) 92 (40%) 21 (9%) 01 (0.4%)	21 (15%) 43 (32%) 61 (45%) 10 (7%) 01 (1%)	20 (22%) 30 (33%) 31 (34%) 11 (12%) 0 (0%)	0.302
Healthcare Coverage	Public - No Cover Public - Medical Card Private Insurance	04 (2%) 105 (53%) 89 (45%)	2 (2%) 46 (56%) 49 (43%)	2 (2%) 41 (49%) 40 (48%)	0.671
Distance to GP	Median, IQR Range < 5 km < 10 km < 15 km ≥ 15 km	3, 1.5-6 0-100 138 (59%) 55 (24%) 23 (10%) 18 (8%)	3, 1.5-6 0–42 82 (58%) 36 (25%) 14 (10%) 10 (7%)	3, 1.25-6 1-100 56 (61%) 19 (21%) 9 (10%) 8 (9%)	0.895 0.846
Distance to ED	Median, IQR Range < 10 km < 25 km < 50 km ≥ 50 km	11.50,5–30 1-160 102 (41%) 62 (25%) 63 (26%) 19 (8%)	17.50 ,6–32 1–90 55 (38%) 36 (25%) 42 (29%) 13 (9)	10, 5-26.5 1-160 47 (47%) 26 (26%) 21 (21%) 6 (6%)	0.085 0.343
Mode of Transport to ED	Private Car Public Transport Other	202 (82%) 34 (14%) 10 (4%)	129 (88%) 12 (8%) 5 (5%)	73 (73%) 5 (3%) 5 (5%)	**0.006**

### General practitioner consultation prior to attendance at the ED

Participants were asked about their decision regarding GP consultation before attending ED and the results showed that of the patients who were referred by a GP to the ED a total of 71% saw their GP in person while 33% had a phone consultation. In this study 2% of patients reported that they did not have a GP, 4% of patients could not contact their GP and 2% saw their GP but were unhappy with their consultation.

### Reasons for ED Attendance

With regard to general reasons for visiting the ED, the majority of respondents (63%) reported that the ED is the “best place” to seek emergency treatment (Table 2) with no significant difference recorded across the two groups (*p* = 0.933). The second most common reason for ED visits was observed as participants being unaware of other available services (27%) and a significant difference was observed across two groups (GP referrals 33% vs. Self-referrals 20%; *p* < 0.05). A significant difference was also observed between the groups regarding awareness of other services open at the time (GP referrals 4% vs. Self-referrals 12%; *p* < 0.05).

**Table 2:** General Reasons for Attendance at the ED (*n* = 249)

General Reasons for ED Attendance	Total *n* = 249	GP referral *n* = 147	Self-referral *n* = 102	*P* value
The ED is the best place for my problem	157 (63%)	93 (63%)	64 (62%)	0.933
I’m unaware of other services to treat me for this problem	68 (27%)	48 (33%)	20 (20%)	**< 0.05**
My family told me to come to the ED	27 (11%)	13 (9%)	14 (14%)	0.223
I don’t know what other services are open at this time	18 (7%)	6 (4%)	12 (12%)	**< 0.05**
It is easy for me to get to the ED	15 (7%)	8 (5%)	7 (7%)	0.643
I attended the ED before, and I was happy with it	13 (5%)	10 (7%)	3 (3%)	0.178
I think I will be seen quicker here than at any other service	15 (6%)	7 (5%)	8 (8%)	0.315
I usually come to ED with a medical problem	11 (4%)	6 (4%)	5 (5%)	0.757
I could not afford to go anywhere else	6 (2%)	3 (2%)	3 (3%)	0.649

In terms of specific reasons for ED attendance, 41% of the respondents considered their presenting condition to be an ‘Emergency’. Participants frequently believed that they might need an x-ray or scan (32%) with almost a third of patients (27%) thinking they may require hospital admission (Table 3). Significant differences were observed across the groups in relation to attending ED due to a need for reassurance at the time of injury/illness (GP referral 32% vs. Self-referral 14%; *p* < 0.001**)** and for the purpose of acquiring a second opinion (GP referral 12% vs. Self-referral 17%; *p* < 0.05).

**Table 3:** Specific Reasons for Attendance at the ED (*n* = 249)

Specific Reasons for ED Attendance	Total *n* = 249	GP referral *n* = 147	Self-referral *n* = 102	*P* value
I consider this condition to be an emergency	102 (41%)	58 (39%)	44 (43%)	0.561
I thought I needed an x-ray or scan	80 (32%)	50 (34%)	30 (29%)	0.444
I thought I might need to go into hospital	67 (27%)	38 (26%)	29 (28%)	0.652
I need reassurance that my illness/injury is not serious	61 (24%)	47 (32%)	14 (14%)	**< 0.001**
I wanted to see a doctor or a nurse as soon as possible	47 (19%)	25 (17%)	22 (22%)	0.366
I came to the ED to get a second opinion	30 (12%)	25 (17%)	5 (5%)	**< 0.05**
I thought I might need a blood test	24(10%)	16 (11%)	8 (8%)	0.424
I wanted to see a specialist	21 (8%)	16(11%)	5 (5%)	0.095
I thought I needed the wound treated	11 (4%)	4 (3%)	7 (7%)	0.118
I thought I might need a tetanus injection	8 (3%)	1 (1%)	7 (7%)	**< 0.05**
I am on a waiting list and I thought this would speed it up	4 (2%)	2 (1%)	2 (2%)	0.711

### Clinical outcomes

The most common clinical presentation to the ED related to Musculoskeletal problems (e.g., Fractures) 26%, followed by Gastrointestinal (e.g., vomiting) and Cardiovascular (e.g., Chest pain) issues recorded as 14% for both categories (Table 4). A total of 11% of participants presented to the ED with complaints of trauma and 7% with respiratory problems. Significant differences were observed between the cases referred by GP and self-referred patients in relation to presenting complaints. For example, differences were observed in relation to patients presenting for trauma (GP referral 16% vs. Self-referral 8%; *p* < 0.05) and for attendances related to gastroenterological problems (GP referral 20% vs. Self-referral 5%; *p* < 0.05).

In the full cohort almost half of the participants (44%) disclosed a duration of presenting complaint for > 7 days while 31% expressed the duration of complaints of < 1 day before presentation to ED. Significant differences were recorded across groups in terms of duration of complaint *p* < 0.001*)*, 50% of the self-referred patients presented to the ED with a duration of complaints < 1 day in comparison to 18% of those who were GP referred. It was observed that there was no statistical difference across the groups regarding Worry levels (Median 7; IQR 5–8; *p* = 0.593) and on the Pain scale (Median 6; IQR 3–8; *p* = 0.678).

There were no statistical differences observed between two groups in terms of triage category (*p* = 0.379), with 56% of cases listed in the “Urgent” triage category and 2% listed in the “non-urgent” category in the full cohort. In relation to LOS approximately a third of patients (34%) remained in the ED for 4–8 h.

Regarding disposition, around two thirds (69%) of participants were discharged home following treatment from ED while 28% of cases were admitted to hospital and 2% left the hospital without waiting for treatment. There was no statistical difference recorded between the two groups for disposition (*p* = 0.434).

**Table 4:** Clinical characteristics of GP referrals and self-referrals to the ED (*n* = 249)

Category	Variable	TOTAL *n* = 249	GP referral *n* = 147	Self-referral *n* = 102	*P* value
Presenting Complaint	Musculoskeletal Gastroenterological Cardiovascular Trauma Respiratory	65 (26%) 35 (14%) 34 (14%) 28 (11%) 17 (7%)	36 (24%) 30 (20%) 21 (14%) 12 (8%) 10 (7%)	29 (28%) 5 (5%) 13 (13%) 16 (16%) 7 (7%)	**< 0.05**
Duration of Complaint	< 1 day 1–2 days 3–7 days > 7 days	77 (31%) 22 (9%) 42 (17%) 108 (44%)	27 (18%) 15 (10%) 32 (22%) 73 (50%)	50 (50%) 7 (7%) 10 (10%) 35 (34%)	**< 0.001**
Worry Scale 1–10	Median, IQR	7, 5–8	7, 5–8	7, 5–8	0.593
Pain Scale 1–10	Median, IQR	6, 3–8	6, 3–8	6, 3–8	0.678
Triage Category	Very Urgent Urgent Standard Non-Urgent	40 (17%) 131 (56%) 59 (25%) 5 (2%)	25 (18%) 84 (59%) 30 (21%) 3 (2%)	15 (16%) 47 (50%) 29 (31%) 2 (2%)	0.379
Length of Stay (LOS)	Median, IQR Range < 1 h 1–2 h 2–4 h 4–8 h 8–16 h 16–24 h > 24 h	5.21,2.5-8 0.02–67 16 (7%) 19 (8%) 61 (26%) 80 (34%) 52 (22%) 8 (3%) 1(0.4%)	5.41,2.53–8.27 0.02–67 9 (6%) 12 (8%) 40 (28%) 43 (30%) 31 (22%) 7(5%) 1 (1%)	5.16,2.5–7.34 0.04–19 7 (7%) 7 (7%) 21 (22%) 37 (39%) 21 (22%) 1 (1%) 0(0%)	0.655 0.486
Disposition	Admitted Discharged Did Not Wait Transferred	62 (28%) 154 (69%) 5 (2%) 1(1%)	42 (31%) 88 (66%) 3 (2%) 1 (1%)	20 (23%) 66 (75%) 2 (2%) 0 (0%)	0.434

### Health services utilisation

Almost half of participants (49%) reported an ED attendance in the previous year and 23% of the participants visited the ED more than once (range 2–7) over the past 12 months (Table 5).

A total of 30% of respondents reported hospital admissions and 39% visited out-patient services in the past year. Out-of-hours (OOH) GP services were also utilised (25%) by the participants and multiple visits (2–6) were recorded by 12% of the patients in the full cohort. Injury units were visited by 9% of the participants over the past 12 months.

In the overall cohort, 90% of the participants reported attending a GP in the past year. There were significant differences observed between the two groups in utilisation of GP services (*p* < 0.05) where 18% of the self-referred patients reported no visit to the GP in the past year when compared to the patients referred by a GP (5%).

**Table 5:** Health Service Utilisation in the last 12 months (*n* = 249)

Health Service	Freq.	TOTAL *n* = 249	Referred by GP *n* = 147	Self-Referral *n* = 102	*P* value
Hospital Admission	0 1 2–3 4–6 7+	171 (70%) 50 (20%) 21 (9%) 3 (1%) 1 (0.4%)	98 (69%) 33 (22%) 12 (8%) 2 (1%) 0 (0%)	73 (72%) 17 (17%) 9 (9%) 1 (1%) 1 (1%)	0.600
Out-Patient Department	0 1 2–3 4–6 7+	151 (61%) 40 (16%) 41 (17%) 7 (3%) 7 (3%)	90 (60%) 24 (17%) 22 (15%) 5 (3%) 4 (3%)	61 (60%) 16 (16%) 19 (19%) 2 (2%) 3 (3%)	0.913
Emergency Department	0 1 2–3 4–6 7+	127 (51%) 62 (25%) 50 (20%) 6 (2%) 2 (1%)	75 (51%) 37 (25%) 29 (20%) 4 (3%) 1 (1%)	52 (51%) 25 (25%) 21 (21%) 2 (2%) 1 (1%)	0.993
Injury Unit	0 1 2–3 7+	186 (91%) 12 (6%) 4 (2%) 1 (1%)	107 (91%) 9 (8%) 1 (1%) 1 (1%)	79 (93%) 3 (4%) 3 (3%) 0 (0%)	0.266
Out-of-Hours (OOH) GP	0 1 2–3 4–6 7+	167 (75%) 28 (13%) 24 (11%) 3 (1%)	96 (73%) 17 (13%) 16 (12%) 1 (1%)	71 (77%) 11 (12%) 8 (9%) 2 (2%)	0.561
General Practitioner	0 1 2–3 4–6 7+	26 (10%) 52 (27%) 87 (35%) 38 (15%) 44 (18%)	8 (5%) 34 (23%) 54 (37%) 25 (17%) 26 (18%)	18 (18%) 18 (18%) 33 (33%) 13 (13%) 18 (18%)	**< 0.05**
Public Health Nurse	0 1 2–3 4–6 7+	211 (86%) 13 (5%) 12 (5%) 4 (2%) 6 (2%)	129 (89%) 6 (4%) 5 (3%) 3 (2%) 2 (1%)	82 (81%) 7 (7%) 7 (7%) 1 (1%) 4 (4%)	0.305
Allied Health Professional	0 1 2–3 4–6 7+	192 (78%) 13 (5%) 25 (10%) 13 (5%) 2 (1%)	114 (79%) 9 (6%) 11 (8%) 9 (6%) 2 (1%)	78 (78%) 4 (4%) 14 (14%) 4 (4%) 6 (6%)	0.303

## Discussion

The aim of this study was to describe the demographic and clinical characteristics of patients who self-refer to EDs in comparison to patients referred by GPs in the Irish setting. According to our findings there were no significant difference between self-referred patients compared to GP referred patients in terms of demographics including; age, gender, civil status, residential status, principal status, socioeconomic status, and distance to a GP surgery. It was also found that healthcare coverage relating to the cost of the ED visit did not differ across the groups. This finding is in-keeping with previous studies suggested that an ED presentation is rarely driven by financial considerations [25].

Nationally it has been reported that annual attendance to a GP is 3.3 consultations on average per patient [26]. This study demonstrated significant differences across the groups regarding GP utilisation rates annually. It was found that 18% of the self-referred patients to the ED had not visited their GP over the past year compared to 5% of the GP referred patients (*p* < 0.05). In Norway most patients admitted to hospital for common acute emergency diagnoses had been in contact with a GPs during the year and in the month prior to the admission [27]. However, a recent study based in London found that infrequent use of the GP surgery was directly associated with low attendance at ED. It may be that lack of access to primary care does not primarily account for ED attendance but rather it is a function of the complex nature of disease and longevity [28].

The analysis shows that a very low number of participants used the services of primary care clinicians requiring GP referral, such as Public Health Nurses and Allied Health Professionals over the past year. These findings provide an important contribution to the ongoing debate on whether patient health can be improved by providing high quality community-based care hence, reducing the burden on EDs [29]. It is unclear whether primary care access can reduce ED visits in the research literature, however, more widely available additional diagnostic facilities outside of hospital could help to reduce ED visits. The World Health Organisation’s overview of integrated care models considers diagnostic imaging in the community as being key to shifting care provision away from hospital-based acute settings [30]. A recent scoping review also concluded that direct access to diagnostic imaging services within General Practice improves system level and individual patient outcomes [31]. In an Irish survey 86% of the GP respondents were of the opinion that access to diagnostics in primary care would reduce their ED referrals and improve their referral quality [32].

The results showed that the majority of participants (63%) chose the ED for their care because they believed that the ED was the best place for their treatment. A previous qualitative study of patient’s choices in using emergency health services described the same findings, that despite easy access to other services (e.g., walk-in and primary care clinics) patients felt these services were unable to meet their needs when perceived as urgent [33]. While acknowledging that walk-in clinics were not operational during certain periods of the COVID-19 pandemic, in this study it is still notable that patients reported another common reason for ED attendance was lack of awareness of other services (27%) and significant differences were observed between the groups regarding the availability of other services open at the time (GP referral 4% vs. Self-referral 12%; *p* < 0.05). This likely reflects the fact that some of the GP referrals had made use of OOH GP services prior to ED attendance and self-referrals may not be aware of these as a care pathway. To decrease ED crowding, awareness is a point of debate and a previous systematic review described those educational interventions that target demand were not effective when an intervention was conducted in a stand-alone manner i.e., when the aim is only to reduce health services utilization [34].

In this study, significant differences were observed across the groups in relation to presenting complaint, however the most frequent clinical presentation to the ED for the full cohort was for musculoskeletal problems (26%), These findings are in accordance with a previous Netherlands based study indicating the most common presentation to ED was also for musculoskeletal problems [35]. In our study almost a third of patients referred by GP to the ED required an x-ray or scan. This suggests that increased awareness of Injury Units and expansion of alternative care pathways (ACP) with appropriate diagnostic facilities in the community, including GP clinics could potentially decrease ED attendance for minor injuries [30, 31]. Access to radiology by out-of-hours GPs in the Netherlands has been reported to reduce referrals to the ED by 39% [36]. However, it must be acknowledged that even with advanced diagnostics in the community some low acuity cases can still warrant hospital attendance as emergent conditions.

A desire for reassurance at the time of injury/illness appeared an important element for the patients referred by a GP with a significant difference observed in comparison to self-referrals (32% vs. 14%; *p* < 0.001). This likely reflects health seeking behaviour, considering that patients referred by a GP in this study were also significantly more likely to have also used other health services in the past 12 months. Previous literature has found that the presence of patient anxiety strongly influenced healthcare seeking behaviour and was associated with reassurance that the patient receives from ED services [9]. GPs attribute a potentially escalating effect to information gathered through the internet and media, and in order to reduce non-urgent ED consultations, health education may hold promise [37]. For example, enhancing educational initiatives for patients to self-manage their care has been shown to be effective in reducing ED visits for older adults and in decreasing admissions for patients with chronic conditions [38].

In terms of duration of complaint, there was a significant difference across groups and this analysis showed that 50% of patients who were referred by a GP had a duration of complaints for > 7 days. For patients with a presenting complaint of < 1 day GP referrals comprised 18% in comparison to 50% of self-referrals (*p* < 0.001). With regard to core ED metrics such as triage, LOS and disposition no significant differences were observed across the groups. In relation to disposition, 31% of GP referrals were admitted compared to 23% of self-referrals and this correlates well with recently reported disposition figures nationally for GP referrals (27%) and self-referrals (23%) [39]. An observational study in Central London previously reported that 49% of hospital admissions related to patients referred by a GP. This suggests that GPs can precisely triage most of the patients who are acute cases and those who need further investigations or specialist investigations [40]. In the Irish setting, previous work from the BDBP study has indicated that achieving consensus between GP and emergency medicine consultants on “appropriate” ED presentations has proved challenging, however when both clinicians deemed an attendance “appropriate” the admission rate was 47% compared to 0% for “inappropriate” attendees [41].

With regard to future research, there is limited data on the decision-making process behind GP referrals to the ED which needs further evaluation. Additional information on the diagnostics and treatment that GP referrals received in the ED could help to assess and contextualise the appropriateness of these referrals. While patient perspectives on ED attendances were included in this study, further qualitative research could investigate ED staff members opinions as to why patients preferentially chose the ED above other care pathways, based on their experiences.

### Limitations

Lower acuity attendances to regional Irish EDs are descriptively analysed in this study over the course of a one-year study; thus, outcomes may not be completely applicable to other settings. Patients attending the ED by emergency ambulance were excluded from this study, however it is the case that GPs also request ambulance transfers from GP clinics, nursing homes and home visits which may mean that GP referrals were underrepresented in the study. This study did not include any mental health or drug/alcohol-related presentations because interviewing a distressed patient poses ethical issues. Another limitation of this study is that patients were excluded who were unable to communicate effectively in English therefore patients from different ethnicities may be under-represented. It was not possible to include non-English language versions of the patient survey as this would require Research Nurses to be multi-lingual which was not feasible in this context. As a result of the COVID-19 pandemic, data collection was delayed, and this was not statistically considered in site comparisons. Sample size is a limitation which may have impacted on statistical significance for some variables which did not have complete datasets. Patients self-reported their proximity to health services (GPs and EDs) and this was not validated by address. This is a limitation of this data considering the known issues surrounding reliability of self-reporting however this basic measure was deemed adequate for this study. Study strengths include capturing patterns of healthcare usage and health seeking behaviours in detail.

## Conclusions

The findings of this multi-site cross-sectional study demonstrate that Irish patients frequently consulted their GP prior to ED attendance during the COVID-19 pandemic. Demographics were similar across groups, however significant differences were observed between GP referrals and self-referrals to the ED in relation to clinical outcomes and health service utilisation rates, These findings may prove useful to policymakers in relation to future service planning generally, and more specifically in relation to patient care during future pandemics.
